# Somatic Care with a Psychotic Disorder. Lower Somatic Health Care Utilization of Patients with a Psychotic Disorder Compared to Other Patient Groups and to Controls Without a Psychiatric Diagnosis

**DOI:** 10.1007/s10488-015-0679-0

**Published:** 2015-09-28

**Authors:** Wilma Swildens, Fabian Termorshuizen, Alex de Ridder, Hugo Smeets, Iris M. Engelhard

**Affiliations:** 1 Altrecht Institute for Mental Health Care, Lange Nieuwstraat 119, 3512 PG Utrecht, The Netherlands; 2 Julius Center for Health Sciences and Primary Care, University Medical Center Utrecht, Heidelberglaan 100, 3584 CX Utrecht, The Netherlands; 3 Division of Pharmacoepidemiology and Clinical Pharmacology, Utrecht Institute for Pharmaceutical Sciences, Utrecht University, PO Box 80082, 3508 TB Utrecht, The Netherlands; 4 Altrecht Institute for Mental Health Care, Gedachtengang 1, 3705 WH Zeist, The Netherlands; 5 Achmea Health Insurance, PO Box 19, 3800 HA Amersfoort, The Netherlands; 6 Department of Clinical and Health Psychology, Utrecht University, Heidelberglaan 1, 3584 CS Utrecht, The Netherlands

**Keywords:** Mental health, Schizophrenia, Somatic Health Care utilization

## Abstract

Patients with non-affective psychotic disorders (NAPD) face higher risk of somatic problems and early natural death compared to the general population. Therefore, treatment guidelines for schizophrenia and psychosis stress the importance of monitoring somatic risk factors. This study examined somatic Health Care utilization (HCu) of patients with NAPD compared to non-psychiatric controls and patients with depression, anxiety or bipolar disorders using a large Health Insurance database. Results show lower specialist somatic HCu of patients with NAPD compared to matched controls and also lower percentages for prescribed somatic medication and general practitioner consultations for patients aged ≥60 years and after longer illness duration.

## Introduction

An increasing body of evidence suggests that patients with non-affective psychotic disorder (NAPD), which involves schizophrenia, schizophreniform disorder, schizoaffective disorder, delusional disorder and psychosis not otherwise specified (Kendler et al. 1996; Kessler et al. 2005) have somatic problems in need of clinical attention. They have a higher risk of physical disorders and a mean life expectancy that is at least 15 years shorter compared to the general population (Altamura et al. 2011; Carney et al. 2006; De Hert et al. 2011a; Wahlbeck et al. 2011; Weber et al. 2009). Premature mortality is strongly associated with natural death causes and the increased relative risk of natural death is already present at a relatively young age (Hennekens et al. 2005; Capasso et al. 2008; Termorshuizen et al. 2013). Associated causes include higher somatic disease incidence (often cardiovascular and respiratory diseases), but a lower quality of somatic health care, underdiagnosis and undertreatment (Bradford et al. 2008; De Hert et al. 2011a; Mitchell et al. 2009; Nasrallah et al. 2006; Laursen et al. 2011). Patients with severe mental illness (SMI) also report more difficulties in accessing care than the general population (Bradford et al. 2008).

Given the poor health condition of patients and increased risk of natural death causes (Laursen et al. 2009; Laursen et al. 2011), the guideline for schizophrenia and other psychotic disorders (NICE 2009) states that a high level of somatic health care is often needed. This guideline stresses the importance of early detection of cardio-vascular risk factors, also referred to as the ‘metabolic syndrome’. It recommends that regular somatic screening is done that covers family disease history and personal risks on diabetes mellitus, hypertension, cardiovascular diseases, cancer, smoking, diet, activity and other metabolic risk factors. Indicated treatment should start as early as possible. It also recommends that general practitioners (GPs) and mental healthcare professionals work closely together to meet the somatic needs of patients with psychosis, who often do not communicate their somatic complaints adequately (De Hert et al. 2011b, c).

Studies have found divergent results with regard to somatic Health Care use (HCu) by individuals with NAPD and other forms of SMI. They typically focused on specific types of HCu. For instance, Carr et al. (2003) found higher patterns of overall *psychiatric and non*-*psychiatric health service* use for patients with psychotic disorders in Australia compared to patients with nonpsychotic disorders. Dickerson et al. (2003) reported mixed findings on HCu, such as higher medical care use and lower dental care use, among outpatients with SMI in Baltimore, Maryland (USA) compared to matched subsets of individuals from the general population. Oud et al. (2010) found that patients with NAPD had more frequent *GP consultations and home visits in general primary care* in The Netherlands, compared to patients with other mental disorders and patients without mental disorders. Studies have also reported similar or lower rates of *specialist HCu* for patients with psychoses or SMI (Laursen et al. 2009). Other research suggests underutilization in HCu, especially in *preventive care and primary care,* compared to controls with and without another psychiatric diagnosis (Druss et al. 2002; Hippisley-Cox et al. 2007; Folsom et al. 2002; Roberts et al. 2007) and in *specialist services* (Salsberry et al. 2005; Young and Foster 2000; Nasrallah et al. 2006).

It should be noted that higher or similar levels of HCu do not preclude serious undertreatment. Domino et al. (2014) found that the quality of care metrics was generally lower among patients with depression or schizophrenia than for other patients in an adult Medicaid population with two or more out of eight chronic conditions (asthma, chronic obstructive pulmonary disease, diabetes, hypertension, hyperlipidemia, seizure disorder, depression, or schizophrenia). In addition, high utilization of emergency rooms for patients with SMI and a higher risk of hospitalization due to ambulatory care-sensitive medical conditions may point to poor access or inefficient use of the primary health care system (Salsberry et al. 2005; Muck-Jorgensen et al. 2000; Li et al. 2008). In fact some studies point to higher levels of undertreatment especially for the most vulnerable subsets of patients such as older or homeless persons with SMI (Young and Foster 2000; Folsom et al. 2002; McCarthy and Blow 2004). Parity in the access to care seems to be hindered by a mixture of patient, provider treatment and system factors. For instance, patients may choose not to seek help for physical problems due to symptoms of SMI, there is a focus on mental rather than physical health problems in mental health care, people with mental disorders are sometimes stigmatized by physicians, and the funding of somatic care throws up financial barriers (De Hert et al. 2011b).

Few large studies so far have compared patients with NAPD to healthy controls and patients with other psychiatric disorders across the full spectrum of HCu (i.e., general and specialist somatic care). Yet, they are important in monitoring possible undertreatment and finding its correlates. Accordingly, the aim of the current study was to examine whether patients with NAPD have a lower somatic HCu level compared to controls without NAPD. We performed a retrospective observational study using linked registry and health plan data to compare HCu by patients with NAPD to HCu by non-psychiatric matched controls. For a sound interpretation, we also included patients with an anxiety, depressive or bipolar disorder as psychiatric references. Patients with these other psychiatric disorders are also at increased risk of premature natural death (Harris and Barraclough 1998; Laan et al. 2011), but may be less socially marginalized and may show less cognitive impairment, which relates to lower HCu (Carr et al. 2003). Consequently, the HCu of these other patient groups (also compared to controls) may more clearly point out the somatic care needs of patients with NAPD. By comparing NAPD patients to controls from the general population as well as other patient groups, we investigated whether undertreatment for somatic problems of psychosis patients was present and what factors determined this undertreatment.

## Methods

### Databases

Since 1999, the Psychiatric Case Registry for the Central Netherlands (PCR-MN) has received anonymous information on patient characteristics and mental health care (MHC) of patients who use psychiatric services in the city of Utrecht and surrounding municipalities. In 2010, the area had over 760,000 inhabitants and formed a representative sample of 5 % of the Dutch population (Smeets et al. 2011). Under the Dutch system, health insurance is compulsory for all residents. Data from PCR-MN were anonymously linked to the Achmea Health Database (AHD), which records payments for medical care made by the largest insurance company in the region. This database includes payments for drug prescriptions delivered by community pharmacists, payments for GP consultations and for Diagnostic and Treatment Protocols (DTPs, in Dutch: ‘Diagnose Behandel Combinatie’) in all treatment settings (e.g. hospital, emergency department, outpatient clinic). A DTP is the insurance claim containing codes for diagnosis and treatment by medical specialists. Each DTP contains four codes: for a certain type of care, care demand, diagnosis, and treatment followed. We used this information to estimate HCu for physical disorders. Dutch privacy law allows use of these data for scientific research under strict conditions in relation to anonymity and storage, in which case informed consent is not needed. The research was approved by the institutional review board.

### Patients and Data Extraction

Data were extracted for all 4770 adult patients (18 years) with NAPD with least one care contact between January 2007 and December 2009 at Altrecht MHC, which is the main mental health service in the Utrecht region. NAPD was defined conform DSM-IV subgrouping (codes 295.10, 295.20, 295.30, 295.40, 295.60, 295.90, 295.70, 297.1, 297.30, 293.81, 293.82, 298.80, 298.90). Half of these patients (2392) were insured with Achmea. This insurance company serves for a relatively large number of insured patients with lower social economic status, similar to Medicaid in the USA. We extracted data from the AHD on the HCu in the year before the last care contact at mental health care service Altrecht in the period 2007–2009 (‘index date’). Controls without NAPD were randomly selected from the AHD and were personally matched: for each individual patient six unique control persons with similar birth year, gender, and ethnic group (Western vs. Non Western) but without NAPD were selected from the AHD (Table 1). We extracted the data on HCu by the controls for the same year prior to the last care contact of the matched patient with a psychiatric diagnosis. We followed the same procedure for other groups with a psychiatric diagnoses: bipolar disorder (N = 700), unipolar depression (N = 5603), anxiety disorder (N = 1707). In the event of psychiatric comorbidity, patients were categorized according to their most severe disorder, which was NAPD, bipolar depression, major depression and anxiety disorder, respectively.

**Table 1:** Study population

	Patients	Matched controls
Total	N = 10,402	N = 61,850
Gender N (%)		
1. NAPD		
Male	1438 (60.1)	8628 (60.1)
Total	2392	14,350
2. Bipolar		
Male	284 (40.6)	1704 (40.6)
Total	700	4200
3. Unipolar		
Male	1958 (34.9)	11,604 (35.1)
Total	5603	33,058
4. Anxiety		
Male	659 (38.6)	3954 (38.6)
Total	1707	10,242
Age Mean (SD)		
1. NAPD	47.8 (14.8)	47.5 (14.9)
2. Bipolar	51.6 (14.1)	51.2 (14.1)
3. Unipolar	46.2 (15.5)	46.1 (15.6)
4. Anxiety	42.2 (14.9)	42.0 (14.9)
Non-Western ethnic origin (%)		
1. NAPD	21.2	21.2
2. Bipolar	6.0	6.0
3. Unipolar	29.4	30.6
4. Anxiety	21.3	21.3
Duration since earliest registered Mean (SD)		
Psychiatric diagnosis		
1. NAPD	5.2 (3.5)	5.2 (3.5)
2. Bipolar	5.1 (3.4)	5.1 (3.4)
3. Unipolar	3.0 (2.9)	3.0 (2.9)
4. Anxiety	2.3 (2.7)	2.3 (2.7)

## Analysis

### Patients with a Psychiatric Diagnosis Versus Controls

HCu by patients in the four mental health categories and by their matched controls was estimated in terms of percentages of patients with ≥1 somatic medication prescription, ≥1 GP consultation, ≥1 somatic specialist consultation, and at least one of these sources of somatic HCu. Using a logistic regression model, we calculated the average marginal effect (AME; Williams 2012) and 95 %-confidence interval (95 % CI) to test the difference in percentages of somatic HCu between the groups of patients with a psychiatric diagnosis and the controls without a psychiatric diagnosis. We also differentiated the HCu in somatic disease category as diagnosed by the specialist (and registered in the DTPs): lung diseases, cardio-vascular diseases, cancer, diabetes or other physical disorders. Presence of a specific prescribed drug (e.g., antiarrhythmics, blood glucose lowering drug) and/or a specific diagnosis in a registered DTP (e.g. ischemic heart disease, diabetic retinopathy) was regarded as presence of somatic care for these physical disorders. Also the average costs reimbursed for the utilization of somatic health services were estimated and the difference (Delta) between patients and controls was tested using a *t* test. Appendix 1 lists somatic diagnoses and information on the Anatomical Therapeutic Chemical classification system used to specify somatic disease categories of HCu.

### Patients with NAPD versus Controls: Determinants of the Difference in HCu

We analyzed the effects of age and duration since earliest registered psychiatric diagnosis and other covariates (gender, ethnic minority status, psychiatric comorbidity) on the difference in somatic HCu between patients with NAPD and their matched controls in a multiple logistic regression model. A significant {covariate × ‘patient vs. control’} interaction indicates that the differences in HCu between patients with NAPD and controls differs across levels of the covariate. To facilitate comparison with other studies on somatic HCu the calculations were also performed for a smaller subset of patients with schizophrenia, schizophreniform and schizoaffective disorder. For each somatic HCu outcome parameter (medication, GP, DTP, and ‘any HCu’) separately, the covariates and terms for interaction were included in one separate multiple logistic model. Finally, we analyzed the difference in somatic HCu between patients with NAPD and controls specifically for cardiovascular diseases and for diabetes.

Data management, record linkage and crude estimations were performed in SPSS 20.0 and regression modeling in STATA 11.0.

## Results

### Study Population

For patients with NAPD in our regional psychiatric case register, 50 % were insured with Achmea during the year prior to index date in 2006–2009. The Achmea insurance rate was lower for the other psychiatric diagnostic groups: 36.1 % for bipolar disorder, 37.0 % for major depression and 31.7 % for anxiety disorders. On average, included patients were older than excluded patients without Achmea database linkage (e.g., for NAPD: 47.8 vs. 43.7 years, *p* < 0.001). They also showed a longer period in care since the first registration of their most severe mental health diagnosis (i.e. the diagnosis used for categorization in this study) in the PCR-MN (e.g., for NAPD: 5.2 vs. 3.6 years, *p* < 0.001).

Table 1 shows characteristics of the study population. More than half of the patients with NAPD were diagnosed with schizophrenia (59.0 %). Schizophreniform, schizoaffective or delusional disorder were also common diagnoses (14.6 %) and 26.4 % suffered from another NAPD, which was often “psychotic disorder not otherwise specified”.

### Patients with a Psychiatric Diagnosis versus Controls: HCu (Univariable)

No significant differences were found between patients with NAPD and their matched controls in the percentages of patients with at least one somatic medication prescription or with at least one GP contact during the year prior to the index date (somatic medication: 72.2 vs. 73.3 %; GP: 77.2 vs. 76.9 %, Table 2). Among patients with bipolar disorder, unipolar depression or anxiety disorder we did find significantly higher percentages of HCu compared to their matched controls. The percentage of patients with NAPD with specialist somatic treatment was even lower compared to their controls (44.3 vs. 47.2 %). Percentages of patients with bipolar, unipolar or anxiety disorders with specialist somatic treatment were all higher compared to their controls. The percentage of patients with NAPD with any somatic HCu (medication and/or GP consultation and/or specialist care) was slightly higher compared to controls [87.3 vs. 85.1 %; AME 2.25 (0.80 to 3.71) *p* = 0.002]. For patients with bipolar disorder, unipolar depression or anxiety disorder, these percentages were markedly higher than for controls, which resulted in large, statistically significant marginal effects (Table 2).

**Table 2:** Somatic Health Care utilization of patients with NAPD, bipolar disorder, unipolar disorder and anxiety, each group compared to matched controls; percentage, average marginal effect (AME)^a^ and (95 %-confidence interval)

Diagnosis	Number of patients/number of controls	% ≥1 medication prescriptions		% ≥1 contacts with general practitioner		% ≥1 DTP with somatic diagnosis		% with any somatic HCu	
		Patients	Controls	Patients	Controls	Patients	Controls	Patients	Controls
1. NAPD	2392/14,350	72.2 %	73.3 %	77.2 %	76.9 %	44.3 %	47.2 %	87.3 %	85.1 %
		AME = −1.12 (−3.05 to 0.82)		AME = 0.36 (−1.46 to 2.18)		AME = −2.89** (−5.04 to −0.74)		AME = 2.25** (0.80 to 3.71)	
2. Bipolar	700/4200	87.6 %	78.4 %	89.3 %	80.2 %	56.1 %	50.2 %	96.1 %	88.6 %
		AME = 9.19*** (6.45 to 11.93)		AME = 9.07*** (6.48 to 11.66)		AME = 5.98**(2.00 to 9.95)		AME = 7.52*** (5.80 to 9.24)	
3. Unipolar	5603/33,058	89.1 %	78.8 %	95.1 %	81.1 %	63.8 %	49.4 %	97.5 %	89.0 %
		AME = 10.24*** (9.31 to 11.17)		AME = 14.09*** (13.38 to 14.79)		AME = 14.46*** (13.09 to 15.83)		AME = 8.48*** (7.95 to 9.01)	
4. Anxiety	1707/10,242	87.6 %	75.3 %	93.6 %	78.6 %	59.9 %	46.1 %	96.7 %	87.0 %
		AME = 12.27*** (10.50 to 14.05)		AME = 14.98*** (13.57 to 16.39)		AME = 13.74*** (11.23 to 16.26)		AME = 9.64*** (8.56 to 10.71)	

*DTP* diagnostic treatment protocol

^a^Difference in percentages of HCu between patients and controls computed as average marginal effect

* p < 0.05, ** p < 0.01, *** p < 0.001

Slightly more pronounced results were found when the analysis for NAPD was restricted to schizophrenia, schizophreniform and schizoaffective disorder (no table included): e.g., the differences for GP contacts and specialist care became larger compared to controls [71.96 vs. 75.47 %; AME 3.52 (−5.85 to −1.18) *p* < 0.01] and [39.76 vs. 45.39 %; AME −5.61 (−8.19 to −3.05) *p* < 0.001], respectively.

### Patients with a Psychiatric Diagnosis Versus Controls: Associated Costs of HCu (Univariable)

**Fig. 1:**
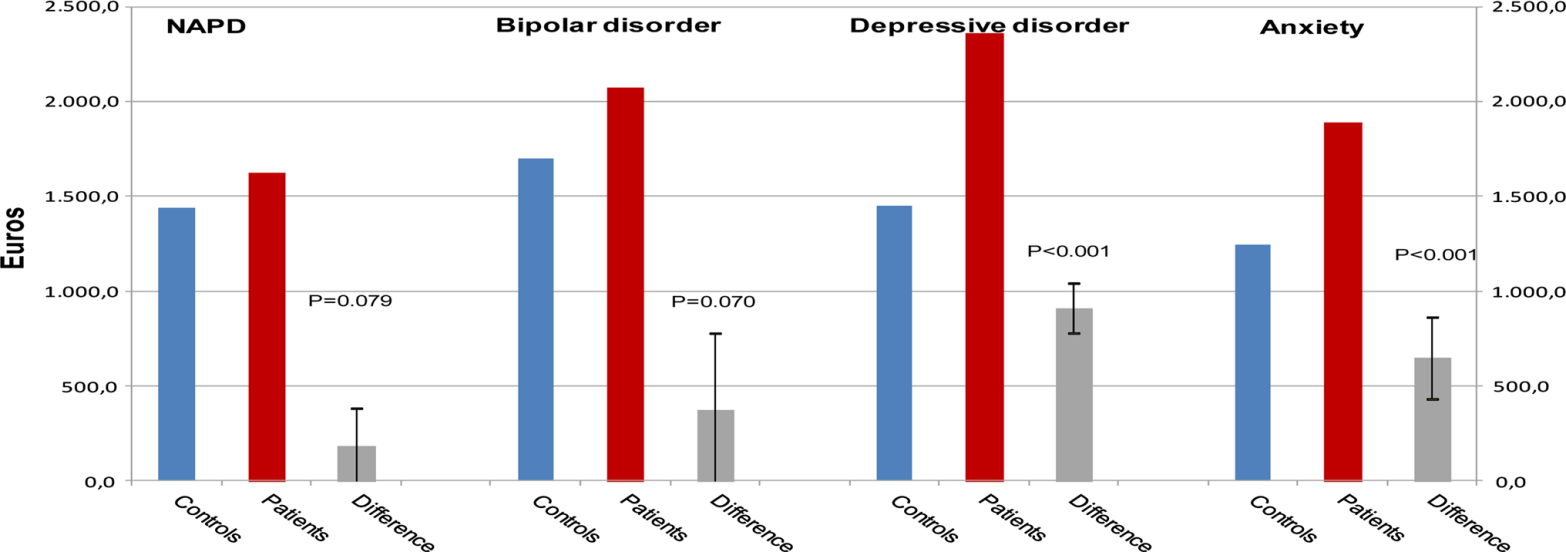
Reimbursed costs associated with Health Care utilization for somatic disorders (medication, GP consults, and specialist care); difference in mean costs (Delta) between patients with NAPD and matched controls and difference in mean costs between patients with other psychiatric diagnoses and matched controls [95 % Confidence Interval]

Figure 1 presents reimbursements for somatic HCu in number of euros per year. Obviously, the average difference in costs for all insurance claims of patients with NAPD compared to their matched controls (Difference, Delta D = €180.30) was lower than the average differences for the other groups with a psychiatric diagnosis compared to their matched controls. Results were similar when we restricted the analysis for NAPD to schizophrenia, schizophreniform and schizoaffective disorder [D = €−94.40 (−327.0 to 137.1)].

For the patients with bipolar disorder, this average difference in costs compared to their matched controls was higher than the difference for the patients with NAPD [D = €373.30 (−30.8 to 777.5) vs. D = €180.30 (−20.9 to 381.6)], but lower than the difference in costs among the patients with depression [D = €910.00 (778.2 to 1041.8)] and anxiety [D = €646.50 (430.6 to 862.4)]. Compared to controls, patients with NAPD did not have significantly higher reimbursements for claims for somatic medication prescriptions [D = €53.40 (−23.6 to 130.5)] or specialist treatment [(D = €90.80 (−77.3 to 259.1)].

### Patients with a Psychiatric Diagnosis Versus Controls: HCu Associated with Treatment by Medical Specialists, Classified into Somatic Diagnosis Categories (Univariable)

Table 3 shows the HCu delivered by medical specialists, as shown in Table 2, but now broken down into somatic diagnostic categories. The relatively low HCu of specialist care by patients with NAPD is mainly explained by lower HCu compared to controls in the following categories: cardiovascular diseases [7.98 vs. 8.51 %; AME = −0.52 (−1.7 to 0.65)], oncology [4.77 vs. 5.66 %; AME = −0.89 (−1.82 to 0.04)] and diabetes [2.88 vs. 3.23 %; AME = −0.35 (−1.08 to 0.38)]. For respiratory disorders, a significantly higher percentage of NAPD patients with a HCu compared to controls was found [5.06 vs. 3.45 %, AME = 1.61 (0.68 to 2.53) *p* < 0.01]. Other diagnosis groups had significantly higher percentages HCu for respiratory disorders compared to controls (AMEs ranged from 2.96 to 3.80), but also for other categories of somatic disorders. Particularly patients with unipolar depression or an anxiety disorder had a higher HCu for cardiovascular diseases compared to their controls [AME = 4.92 (3.99 to 5.85) and 5.95 (4.30 to 7.59) respectively]. We found similar results when we restricted the analysis for NAPD to schizophrenia, schizophreniform and schizoaffective disorder (data available on request).

**Table 3:** Somatic Health Care utilization of patients with NAPD, bipolar disorder, unipolar disorder and anxiety compared to matched controls; percentage, average marginal effect (AME)^a^ and [95 %-confidence interval]

Diagnosis	% ≥1 DTP with somatic diagnosis: Respiratory disorders		% ≥1 DTP with somatic diagnosis: Cardiovascular disorders		% ≥1 DTP with somatic diagnosis: Oncology		% ≥1 DTP with somatic diagnosis: Diabetes		% ≥1 DTP with somatic diagnosis: Other physical disorders	
	Patients	Controls	Patients	Controls	Patients	Controls	Patients	Controls	Patients	Controls
1. NAPD	5.06 %	3.45 %	7.98 %	8.51 %	4.77 %	5.66 %	2.88 %	3.23 %	38.8 %	41.2 %
	AME = 1.61** (0.68 to 2.53)		AME = −0.52 (−1.70 to 0.65)		AME = −0.89 (−1.82 to 0.04)		AME = −0.35 (−1.08 to 0.38)		AME = −2.36 * (−4.47 to −0.25)	
2. Bipolar	7.29 %	4.17 %	9.57 %	10.93 %	7.00 %	7.55 %	3.00 %	3.43 %	50.9 %	42.8 %
	AME = 3.12** (1.10 to 5.14)		AME = −1.36 (−3.73 to 1.02)		AME = 0.55 (−2.60 to 1.50)		AME = −0.43 (−1.81 to 0.95)		AME = −8.05*** (−4.05 to 12.04)	
3. Unipolar	7.10 %	3.30 %	13.05 %	8.12 %	6.03 %	5.52 %	3.59 %	3.10 %	58.4 %	43.5 %
	AME = 3.80*** (3.10 to 4.50)		AME = 4.92*** (3.99 to 5.85)		AME = 0.51 (−0.16 to 1.18)		AME = 0.48 (−0.04 to 1.01)		AME = 14.90*** (13.50 to 16.29)	
4. Anxiety	5.98 %	3.02 %	12.65 %	6.71 %	4.92 %	4.73 %	2.11 %	2.48 %	53.7 %	41.2 %
	AME = 2.96*** (1.79 to 4.13)		AME = 5.95*** (4.30 to 7.59)		AME = 0.19 (−0.91 to 1.30)		AME = −0.37 (−1.11 to 0.37)		AME = 12.49*** (9.94 to 15.04)	

DTP diagnostic treatment protocol

^a^Difference in percentages of HCu between patients and controls computed as average marginal effect

* p < 0.05, ** p < 0.01, *** p < 0.001

### Patients with NAPD Versus Controls: Determinants of the Difference in HCu (Multiple)

The difference in somatic HCu between patients with NAPD and their matched controls differed significantly by strata defined on the basis of age and duration since earliest registration of NAPD in the PCR-MN (3 age categories × 3 duration categories give a factor with nine levels) (Tables 4, 5). Estimates were adjusted for gender, ethnic origin, presence of comorbid depressive and/or personality disorder and/or comorbid substance abuse/dependence. The effect of the interaction with df = 8 for each indicator of HCu refers to the differences between the nine different average marginal effects per stratum, simultaneously tested as one cluster of terms for the 1st order interaction between the above mentioned factor and ‘patient versus control’ in one analysis step. The highly significant test for interaction (*p* < 0.001) does not point to undertreatment in one specific subgroup of patients compared to their matched controls, in the first place, but does show clear differences between categories defined on the basis of age and illness duration. The effect for the indicator ‘any somatic HCu’ presented in Table 5 was a 3.64 percentage point difference (−3.07 to 10.34) for those under 40 and for shorter durations (<2 years), and was substantially and significantly lower (i.e. larger in the negative direction) for older patients ≥ 60 years) with a longer mental health care duration (>5 years): a −10.99 percentage point difference (−17.02 to −4.97) *p* < 0.001.

**Table 4:** Differences in Health Care utilization for somatic disorders of patients with NAPD compared to matched controls by stratum of age and duration since earliest date of registered diagnosis; percentage, average marginal effect (AME)^a^ and 95 % confidence interval

Diagnosis	Number of patients/number of controls	% ≥1 medication prescriptions Patients with NAPD versus controls				% ≥1 contacts with GP Patients with NAPD versus controls			
		%	%	AME	95 % CI	%	%	AME	95 % CI
Age & duration									
<40 years									
<2 years	223/1320	59.1	65.5	−6.32	−14.55 to 1.90	84.9	73.5	11.55***	5.38 to 17.72
2–5	222/1314	64.5	65.2	−0.63	−8.93 to 7.67	74.3	71.0	3.36	−4.31 to 11.02
≥5	384/2304	61.0	66.7	−5.95	−13.21 to 1.31	71.6	73.3	−1.84	−8.80 to 5.11
40–60 years									
<2 years	203/1224	69.3	75.5	−6.24	−14.17 to 1.68	86.3	77.6	8.72**	2.75 to 14.69
2–5	294/1770	66.1	76.0	−9.80**	−16.73 to −2.87	73.0	77.0	−3.95	−10.55 to 2.65
≥5	567/3390	67.8	75.0	−7.31*	−13.05 to −1.57	73.3	77.8	−4.60	−10.20 to 0.99
>60 years									
<2 years	154/934	80.6	87.3	−5.87	−12.56 to 0.82	86.8	84.9	1.71	−4.00 to 7.43
2–5	123/750	69.4	86.2	−15.22**	−23.85 to −6.60	77.4	82.3	−4.50	−12.40 to 3.39
≥5	222/1344	65.9	86.3	−19.05***	−25.97 to −12.12	70.4	84.0	−12.77***	−19.53 to −6.01
Effect of interaction between stratum (age & duration) and patient versus control (df = 8)				p < 0.001				p < 0.001	

Adjusted for gender, ethnic minority, depression, personality disorder, alcohol and drug abuse/dependence

^a^Difference in percentages of HCu between patients and controls computed as average marginal effect

* p < 0.05, ** p < 0.01, *** p < 0.001

**Table 5:** Differences in Health Care utilization for somatic disorders of patients with NAPD compared to matched controls by stratum of age and duration since earliest date of registered diagnosis; percentage, average marginal effect (AME)^a^ and 95 % confidence interval

Diagnosis	Number of patients/number of controls	% ≥1 DTP with somatic diagnosis Patients with NAPD versus controls				% with any somatic HCu Patients with NAPD versus controls			
		%	%	Average marginal effect	95 % CI	%	%	Average marginal effect	95 % CI
Age & duration									
<40 years									
<2 years	223/1320	37.4	38.6	−1.15	−8.89 to 6.59	85.2	81.7	3.64	−3.07 to 10.34
2–5	222/1314	38.1	38.2	−0.02	−7.90 to 7.86	85.6	81.2	4.54	−2.25 to 11.33
≥5	384/2304	34.4	37.8	−3.39	−9.74 to 2.97	80.5	81.9	−1.53	−8.43 to 5.35
40–60 years									
<2 years	203/1224	45.1	47.9	−2.80	−11.01 to 5.41	87.9	86.1	1.18	−4.29 to 7.91
2–5	294/1770	33.2	47.4	−14.27***	−20.87 to −7.67	80.6	86.5	−5.79	−12.1 to 0.55
≥5	567/3390	36.9	46.6	−9.71**	−15.18 to −4.24	81.6	86.1	−4.68	−10.07 to 0.71
>60 years									
<2 years	154/934	58.4	66.2	−7.60	−16.20 to 0.98	91.6	92.5	−0.70	−5.49 to 4.09
2–5	123/750	58.9	65.7	−6.66	−16.27 to 2.94	88.5	91.2	−2.23	−8.44 to 3.98
≥5	222/1344	47.5	64.2	−16.61***	−24.10 to −9.12	80.6	93.1	−10.99***	−17.02 to −4.97
Effect of interaction between stratum (age & duration) and patient versus control (df = 8)				p = 0.0047				p < 0.001	

Adjusted for gender, ethnic minority, depression, personality disorder, alcohol and drug abuse/dependence

^a^Difference in percentages of HCu between patients and controls computed as average marginal effect

* p < 0.05, ** p < 0.01, *** p < 0.001

Similar trends by age and the duration in mental health care were found and significant tests for interaction were obtained for the other indicators of HCu: ‘somatic medication’, ‘GP consultations’ and ‘medical specialist treatment’ separately. Again similar trends were found when we restricted these analyses to schizophrenia, schizophreniform and schizoaffective disorder (data available on request).

Male gender, presence of comorbid depressive disorder, and comorbid alcohol abuse/dependence were associated with a higher somatic HCu among patients with NAPD versus their controls—percentages, AMEs and *p* values for interaction are respectively: 85.21 versus 80.57 %; AME = 5.14 (2.56 to 7.73) *p* < 0.001; 92.99 versus 85.49 %; AME = 6.80 (4.14 to 9.47) *p* = < 0.001; 92.10 versus 85.57 %; AME = 8.60 (4.65 to 12.54) p < 0.001. The percentage of patients with prescribed somatic medication tended to be lower for patients with NAPD versus matched controls (66.86 vs. 72.42 %) when there was a comorbid diagnosis of drug abuse/dependence but this difference tested as AME was non-significant.

### Patients with NAPD vs. Controls: Determinants of Difference in HCu for Cardiovascular Disorders and Diabetes (Multiple)

The somatic HCu related to cardiovascular disorders (i.e. having received cardiovascular medication and/or specialist treatment with cardiovascular diagnosis) among patients with NAPD compared to their matched controls was lower for older patients (≥60 years) with longer durations of their disorder (>5 years) (AME −23.95 [−31.17 to −16.73], p < 0.001). Higher AMEs were found for those patients under 40 with shorter durations (<2 years) compared to controls [AME 3.42 (−0.76 to 7.60)]; p value for 1st order interaction of age and duration <0.001).

Effects of age and duration on the differences in percentages of somatic HCu related to diabetes showed a similar pattern (p value for 1st order interaction = 0.0020). Compared to their age-matched controls, patients over the age of 60 with a longer duration of their disorder received less anti-diabetic medication and/or less specialist treatment for diabetes [AME = −2.76 (−7.98 to 2.45)] whereas higher AMEs were found for younger patients (see Appendix 2).

## Discussion

In this study, we found a slightly higher somatic HCu and associated costs among patients with NAPD compared to their matched controls. However, this difference in HCu was lower than the difference found for other groups with a psychiatric diagnosis, especially a depressive or an anxiety disorder. In addition, the somatic HCu of patients with NAPD was lower compared to their matched controls when the analysis was restricted to treatments delivered by a medical specialist. There was a clear and significant trend toward a negative difference in HCu with increasing age and longer duration since registered diagnosis.

Younger patients with NAPD who spend less time in mental health care had a higher percentage of contacts with the GP compared to controls, but not for specialist care. The comparatively low HCu among patients with NAPD at higher ages was especially noteworthy for cardiovascular diseases and diabetes. Our findings contrast with the poorer health conditions and substantially higher risk of cardiovascular and other early natural death causes of patients with NAPD, as reported earlier. This study therefore strongly suggests underutilization of somatic HC by patients with NAPD.

### Comparison with Other Studies

Our results are consistent with studies in various countries showing that patients with SMI or a psychotic disorder make less overall use of somatic health care than other patients with a psychiatric diagnosis and have a lower HCu of specialist services than patients in the general population. Our findings contrast with the studies from Dickerson et al. (2003) and Carr et al. (2003) who found higher HCu for patients with schizophrenia than for the general population. A possible explanation is that these studies concerned a selection of patients currently receiving outpatient care that may promote attention to physical health.

In our study especially older patients with a longer duration in mental health care had a lower HCu which is also consistent with other research. Notably the research by Folsom et al. (2002) indicates that middle-aged and older homeless people with schizophrenia in California receive less primary and preventive health care and are treated for fewer chronic medical problems than a comparison group with major depression. In the Dutch study by Oud et al. (2010) patients with psychosis had more frequent GP consultations compared to other primary care patients, but similar or lower rates of GP contacts were found, especially among the elderly patients with psychosis, for diabetes, cardiovascular and respiratory diseases. This study was restricted to those with at least one GP consultation during the study period, which may imply that psychosis patients with comparatively high levels of HCu were slightly overrepresented. A higher level of undertreatment at higher ages has also been observed for cardiovascular procedures after myocardial infarction in patients with SMI, especially those with schizophrenia (Young and Foster 2000). Among 142,055 Veterans Affairs patients with SMI, McCarthy and Blow (2004) found that patients over 65 were substantially more negatively affected by distance barriers to use non-psychiatric somatic care. Furthermore, in the study of Laursen et al. (2011) in Denmark, slightly higher rates of heart disease admissions were found among patients with schizophrenia and bipolar disorder, alongside an excess of cardiac mortality. Consistent with our results, they found an interaction between lower admission rates and higher ages. However our findings contrast with the study from Cradock-O’Leary et al. (2002) using data from the Department of Veterans Affairs. They found fewer medical visits and the lowest rates of diagnosed pulmonary disease, diabetes and hypertension among patients with a diagnosis of schizophrenia or bipolar disorder compared with other VA patients. In contrast to our findings, however, a slightly higher number of medical care visits were found at higher ages among those with schizophrenia while younger patients with schizophrenia had an especially high risk of not receiving general medical services. This need for somatic HCu among the partly institutionalized elderly with SMI may come to light more in a comprehensive system of both medical and mental health care such as the Department of Veterans Affairs.

### Strengths and Limitations

Our study has several strengths. We reported HCu for a large group of patients with NAPD, other groups with a psychiatric diagnosis and their matched controls. We examined several aspects of HCu (somatic medication prescriptions, GP and somatic specialist consultations) broken down into specific somatic diagnoses and associated costs. Objective data from insurance claims were used, thus precluding the risk of recall bias. In addition, important covariates—age, gender, psychiatric diagnosis, ethnicity and alcohol and drug dependency—were taken into account. Therefore, this study can contribute to tracking patient groups at high risk of undertreatment.

A number of limitations of the study needs to be addressed. First, the analyses were based on insurance payments for GP consultations and somatic health care treatments. No information was available on the actual presence or severity of somatic disorders, whether diagnosed and treated or not. Second, the data specified the distribution of crude categories of HCu (i.e. medication, GP or specialist care). Administrative database studies like this study can signal general health problems that should be followed by further research. Detailed information concerning duration of admissions and outpatient care, use of specific cardiovascular procedures and/or other preventive measures and degree of integration of the care system could provide more insight into the nature of the somatic underconsumption by patients with NAPD and ways to improve this.

A third limitation is the cross-sectional study design, which was restricted to patients in an arbitrary year prior to the date of the last mental health care contact in 2007-2009. Selection of those who do relatively well and have survived the high mortality risks during the early phases of their treatment (‘healthy survivor’ effect) may lead to an underestimation of HCu (Oud et al. 2010; Laursen et al. 2009). In addition, people who receive mental health care for a longer period may have developed better self-management skills for somatic problems that tie in closely with their experience in managing their psychiatric disorder (Dixon et al. 2004). However these considerations do not fully explain why HCu by patients with NAPD is lower than that of other psychiatric patient groups, who probably experienced similar survival benefits and developed similar self-management skills.

A final limitation is that the aim of the study was to examine whether patients with NAPD have lower levels of somatic HCu compared to psychiatric and healthy controls, but no explanation can be given for the outcomes based on the present results. The in comparison with controls higher percentages with at least one contact with the GP in younger patients with a shorter illness duration since the first registration of their diagnosis in the PCR-MN may point to the important function of the GP in the early diagnostic phases of a psychotic disorder. The observed trend towards lower percentages of contacts with the GP and somatic specialists (compared to age-matched controls) among the elderly NAPD patients with longer illness duration may be partly a result of the aforementioned selection of relatively fit people at higher ages (‘healthy survivor’ effect). However an additional explanation could be that finding your way to the somatic health care system is extra difficult for the most vulnerable patients (Hert, Cohen, et al. 2011b; Thornicroft et al. 2007). Doctors prescribing antipsychotics do not always accurately monitor somatic complications (Okkels et al. 2013), they may find this too stressful for patients with psychosis (Cahn et al. 2008). Reluctance to address patients and distance barriers as found by McCarthy and Blow (2004) could promote somatic undertreatment particularly in older patients. Differences in the distribution of NAPD sub-diagnoses among the age categories is a less likely explanation, as similar results were found when the analysis for Tables 4 and 5 was restricted to schizophrenia, schizophreniform and schizoaffective disorder.

## Conclusions

Our data indicate undertreatment for physical disorders among patients with NAPD particularly for older patients with NAPD. This implies that the national context with compulsory insurance for all inhabitants in the Netherlands does not lead to parity in HCu. The lower HCu and lower costs incurred for medical specialist care among persons with NAPD are reasons for concern. Our data suggest that we have not yet achieved the goals of lowering the cardio-vascular risk factors as formulated in the international guidelines on psychosis and schizophrenia. Improving awareness of the risks of undertreatment among health professionals and the public is an important starting point in bridging the health care gap between patients with SMI and the general population (Thornicroft et al. 2007; Ahire et al. 2013). We suggest a more assertive approach in the way somatic health care is delivered to these patients. By enhancing early detection of risk factors for diseases such as hypertension, weight gain and elevated cholesterol, it may be possible to decrease the rates of early deaths in patients with NAPD. Integration of medical and psychiatric care and preventive interventions may also result in improved quality of medical care (Nasrallah et al. 2006; Druss et al. 2001; Cahn et al. 2008; De Hert et al. 2011b, c). Further studies on somatic health care to patients with NAPD - from the appearance of initial symptoms to help-seeking, assessment, start of treatment and follow-up—are needed to find entry points for improving the match between needs and delivery of treatment.

## Figures and Tables

**Fig. 2:**
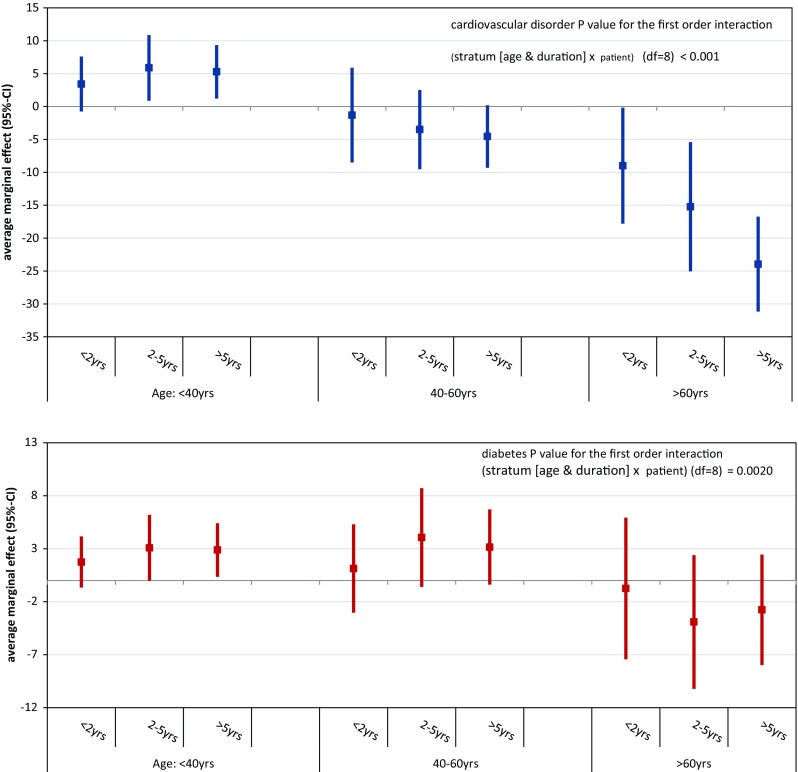
Average marginal effect (95 % CI) of any HCu for cardiovascular disorders and diabetes among persons with NAPD compared to controls matched by age and duration since registered NAPD diagnosis. Adjusted for gender, ethnic minority status, depression, personality disorder, alcohol and drug abuse and/or dependence

## Appendix 1

Information on the diagnosis by medical specialists and prescribed medications that were decisive for inclusion of patients in the categories respiratory disorders, cardiovascular disorders, oncology, diabetes or other physical disorders.

### Respiratory disorders

Included are all respiratory disorders diagnosed by a medical lung specialist [airway diseases such as asthma, emphysema, bronchiectasis and chronic bronchitis, lung tissue diseases such as pulmonary fibrosis and sarcoidosis, unspecified problems (like chest pain, dyspnea, cough) and other diagnoses (such as hyperventilation syndrome, nicotine addiction)] and medications prescribed by specialists or GPs for respiratory disorders (WHO site http://www.whocc.no/, selection of all ATC codes starting with ‘R’).

### Cardiovascular disorders

Included are all cardiovascular diseases diagnosed by a cardiologist (coronary artery disease, heart attack, abnormal heart rhythms or arrhythmias, heart failure, heart valve disease, congenital heart disease, vascular diseases) and medications prescribed by specialists or GPs for cardiovascular disorders (WHO site http://www.whocc.no/, selection of all ATC codes starting with ‘C’).

### Diabetes

Included are the diagnosis of diabetes mellitus (I and II) diagnosed by a medical specialist and medications prescribed by specialists or GPs for diabetes (WHO site http://www.whocc.no/, selection of all ATC codes starting with ‘A10’).

### Oncology

Included are all oncological diagnoses by a medical specialist; breast cancer, skin cancer, lung cancer, colon cancer, prostate cancer, lymphoma and all other (>100 types of) cancers. No extra medication search in the health database was needed since patients never receive cancer medication without a registered diagnosis by a medical oncologist or receive this medication prescribed by their GP.

### Other

All other somatic diagnoses by a medical specialist not included above.

## Appendix 2

See (Fig. 2).
